# Oxygen‐mediated plasticity confers hypoxia tolerance in a corallivorous polychaete

**DOI:** 10.1002/ece3.5929

**Published:** 2020-01-19

**Authors:** Noelle M. Lucey, Mary Collins, Rachel Collin

**Affiliations:** ^1^ Smithsonian Tropical Research Institute Balboa Ancon Panama

**Keywords:** branchial filaments, gill morphology, *Hermodice*, ocean deoxygenation, respiration

## Abstract

There is mounting evidence that the deoxygenation of coastal marine ecosystems has been underestimated, particularly in the tropics. These physical conditions appear to have far‐reaching consequences for marine communities and have been associated with mass mortalities. Yet little is known about hypoxia in tropical habitats or about the effects it has on reef‐associated benthic organisms. We explored patterns of dissolved oxygen (DO) throughout Almirante Bay, Panama and found a hypoxic gradient, with areas closest to the mainland having the largest diel variation in DO, as well as more frequent persistent hypoxia. We then designed a laboratory experiment replicating the most extreme in situ DO regime found on shallow patch reefs (3 m) to assess the response of the corallivorous fireworm, *Hermodice carnaculata* to hypoxia. Worms were exposed to hypoxic conditions (8 hr ~ 1 mg/L or 3.2 kPa) 16 times over an 8‐week period, and at 4 and 8 weeks, their oxygen consumption (respiration rates) was measured upon reoxygenation, along with regrowth of severed gills. Exposure to low DO resulted in worms regenerating significantly larger gills compared to worms under normoxia. This response to low DO was coupled with an ability to maintain elevated oxygen consumption/respiration rates after low DO exposure. In contrast, worms from the normoxic treatment had significantly depressed respiration rates after being exposed to low DO (week 8). This indicates that oxygen‐mediated plasticity in both gill morphology and physiology may confer tolerance to increasingly frequent and severe hypoxia in one important coral predator associated with reef decline.

## Graphical abstract

There is mounting evidence that the deoxygenation of coastal marine ecosystems has been underestimated, particularly in the tropics. We provide clear evidence that severe diurnal hypoxia on shallow coral reefs is associated with continuous hypoxia trends found in deeper bay water. Additionally, we experimentally show that under hypoxia, the fireworm Hermodice carunculata is able to regenerate significantly larger gills compared to worms under normoxia, as well as rapidly recover metabolically after hypoxia exposure.

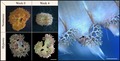

## 1 INTRODUCTION

Most living marine organisms need oxygen to survive. This becomes a problem when there is little dissolved oxygen (DO), or a limited availability of oxygen (pO_2_) in the water. Despite this, hypoxia is commonly encountered in many marine environments (Schmidtko, Stramma, & Visbeck, 2017). In coastal ecosystems, hypoxia is often defined as oxygen concentrations <2 mg/L, or pressures <6.1 kPa (Hofmann, Peltzer, Walz, & Brewer, 2011), and the number of documented sites meeting these criteria has exponentially increased in the last 70 years (Breitburg et al., 2018; Vaquer‐Sunyer & Duarte, 2008). Oxygen levels are predicted to worsen with hypoxic areas extending spatially and persisting for longer time periods, highlighting the need for additional scientific attention focused on understanding the impacts of these changes (Bopp et al., 2013; Breitburg et al., 2018; Schmidtko et al., 2017).

Hypoxia is generally associated with increased nutrients, warming and decreased circulation/stratification (see Altieri & Witman, 2006; Diaz & Rosenberg, 1995 for reviews) causing major shifts in species composition, diversity and general restructuring of ecosystems, usually via local mass extinctions, often followed by recolonization by invasive opportunistic species (Altieri & Witman, 2006; Diaz, 2001; Laboy‐Nieves et al., 2001). However, our understanding of the extent of both the physical changes in ocean deoxygenation and the corresponding biological responses are still in their infancy (Pörtner et al. 2014). This is especially true for tropical systems (Altieri et al., 2017).

In addition to anthropogenic hypoxia, there are also areas with naturally low oxygen concentrations, or natural periodically low oxygen concentrations that have occurred for thousands of years (i.e., upwelling areas, fjords, oxygen minimum zones (OMZs)) (Childress & Seibel, 1998; Gray, Wu, & Or, 2002; Wong, Drazen, Callan, & Korsmeyer, 2018). Marine organisms from chronically hypoxic environments have found mechanisms to cope with this oxygen challenge. The most widely encountered strategy is for organisms to increase the effectiveness of oxygen uptake (Childress & Seibel, 1998; Richards, 2011). This includes both physiological modifications that increase oxygen extraction from the environment (e.g., gill surface area, ventilation rate) and alterations to the oxygen transport system within the body (e.g., hematocrit, Hb–O_2_ binding affinity), as well as potential changes in metabolic demands (Levin, 2003). For example, organisms that can increase their gill surface area are directly increasing the capacity for gas exchange with the environment, therefore compensating for reduced oxygen levels. Amepliscid amphipods and many marine polychaetes (i.e., spionid, dorvilleid, and lumbrierid spp.) have elongated, proliferated and numerous branchiae (gills) and appear to be adapted to permanent hypoxia (Lamont & Gage, 2000). Increased gill surface area is also seen in mysids, fish, and cephalopods from OMZs (Childress & Seibel, 1998). While increased gill surface area is directly linked to increased oxygen uptake, it is also associated with higher ventilation rates, circulation capacity, and blood pigment affinity for oxygen (Childress & Seibel, 1998; Lamont & Gage, 2000; Levin, 2003). These types of responses have primarily been observed in species inhabiting chronic hypoxia and are thought to be evolutionary adaptations to relatively stable low DO conditions (Levin, 2003). Complementary studies on intermittent hypoxia have documented similar modifications, such as increased gill growth in European grass shrimp from salt marsh habitats with severe daily oxygen fluctuations (Peruzza et al., 2018). These few studies additionally suggest that traits developing after short bouts of severe intermittent hypoxia can be quite different to those found in chronic hypoxia exposures and may have more to do with the species' recovery period rather than persistence through hypoxic periods (Borowiec, Darcy, Gillette, & Scott, 2015; Peruzza et al., 2018).

Many coastal areas experience large fluctuations in oxygen that are driven by community metabolism (Diaz & Rosenberg, 1995; Levin et al., 2009). This appears to be particularly true in highly productive systems such as coral reefs (Al‐Horani, Tambutté, & Allemand, 2007; Nelson & Altieri, 2019), which experience large diurnal oxygen fluctuations (Nilsson & Ostlund‐Nisson, 2003; Wong et al., 2018). The organisms inhabiting reefs with this type of diurnal DO regime will experience oxic conditions during the day when photosynthesis drives DO up (either supersaturated or normoxic), and hypoxic conditions at night when respiration dominates (Wild, Niggl, Naumann, & Haas, 2010). Yet, little is known about how the magnitude and duration of such oxygen fluctuations vary in tropical coral reef ecosystems, nor the relative importance of biological and physical processes in determining oxygen availability (see review by Nelson & Altieri, 2019 and references within). As such, the physiological responses of organisms from these systems both within and outside the normal range of oxygen variability are also poorly understood. Plasticity could play a major role in conferring resilience and survivorship under current and future hypoxia circumstances (Botero, Weissing, Wright, & Rubenstein, 2015; Schaum, Rost, & Collins, 2016). If coastal hypoxia is repetitive and predictable, it may promote plasticity, strengthening coastal marine inhabitants' capacity for hypoxia tolerance (Nilsson & Ostlund‐Nisson, 2003; Seebacher, White, & Franklin, 2015; but also see: Clark & Gobler, 2016). Therefore, we aimed to determine the capacity of an important coral predator to show a plastic response to repetitive, yet extreme oxygen changes.

To investigate this idea, we focused on *Hermodice carunculata,* a widely distributed tropical polychaete worm abundant in both dead and live coral‐dominated areas (Yáñez‐Rivera & Salazar‐Vallejo, 2011). This species is found on reefs with documented hypoxia (Nelson, Kuempel, & Altieri, 2016) and appears to thrive in degraded ecosystems that have recently experienced a phase shift to algal dominated communities (Wolf, Nugues, & Wild, 2014). Recent research has also identified worms with fewer gills from the more oxygenated Mediterranean Sea, compared to worms in the West Atlantic, suggesting that gill abundance is a function of environmental oxygen conditions in this species (Ahrens et al., 2013). Our objectives in this study were to document the spatial and temporal variation in hypoxic conditions in a tropical bay where *H. carunculata* is common. We then used this knowledge to simulate different environmental conditions in the laboratory to determine whether the fireworm *H. carunculata* gill morphology changed in response to different DO conditions, and whether these responses were associated with changes in respiratory performance.

## 2 MATERIALS & METHODS

### 2.1 Study site

Bahia Almirante in Bocas del Toro province, Panama is a large bay (446 km^2^) that hosts the second largest (~88 km^2^) reef area on Panama's Caribbean coast (D'Croz, Del Rosario, & Gondola, 2005; Suman & Spalding, 2018). Together with seagrass meadows, these two ecosystems make up the majority of benthic habitats around the archipelago of large islands and mangrove keys (Suman & Spalding, 2018). Depths reach 20–50 m, however, most reefs are found between 2 and 10 m (Guzmán & Guevara, 2001).

The known history of documented hypoxia in the bay began in 2010 when deoxygenated water shoaled from depth to 10 m and persisted in a highly stratified water column, causing death to coral reefs below that depth (Altieri et al., 2017). Subsequent to this, the Smithsonian Tropical Research Institute (STRI) began monitoring the DO concentrations in the bay weekly. We augmented these temporally course data with more detailed, but more short‐term observations to understand the frequency, spatial extent, and persistence of hypoxia in the bay.

### 2.2 Spatiotemporal variation in environmental oxygen concentrations

**Figure 1:**
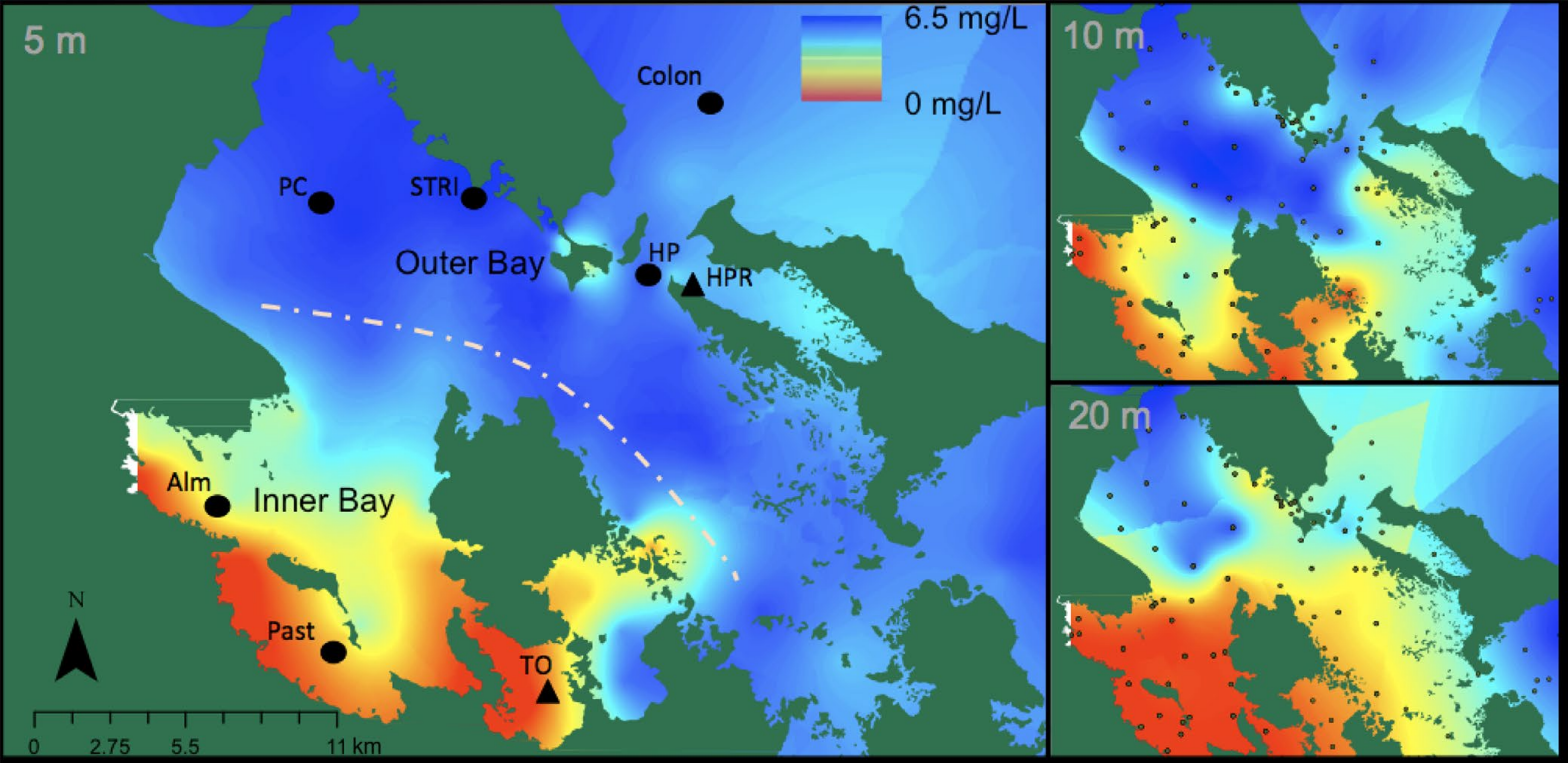
Spatial distribution of DO in Almirante Bay at approx. 5, 10, and 20 m depth on 26 September 2017. Left map shows weekly monitoring sites (dots) and continuous monitoring sites (triangles) at 5 m depth. Right: Maps of DO concentrations at 10 m (top) and 20 m (bottom) depth. Gray points indicate where sites from spatial survey that were used to interpolate values displayed on the three maps. DO range: 0.19–6.76 mg/L; *p*O_2_: 0.629–22.617 kPa; temperature: 29.78–32.56**°**C; salinity: 33.35–36.97 psu

The spatial extent of hypoxia in Bahia Almirante was surveyed by recording depth profiles of DO throughout the water column using a YSI multiparameter sonde with an optical DO sensor (YSI EXO2 & EXO optical DO Smart Sensor) on 26 September 2017, at 83 sites. DO was recorded close to the surface (5 m), at an intermediate depth (10 m), and near the seafloor (<20 m) throughout the bay (Figure 1). Sampling for these data occurred between 8:00 and 17:30 on the same day using two boats, each sampling sites along the DO gradient to minimize and standardize temporal error as much as possible. Daily variations in DO may have resulted in overestimated DO values in the outer bay compared to the inner bay, as these sites were primarily measured in the afternoon. Data points were interpolated with Kriging methods using ArcGIS to visualize the variation in DO on this day, during the hypoxic season.

To understand when the hypoxic season occurs and which depths are affected, we analyzed DO data collected weekly from September 2010 to 2019 by the STRI Bocas station's monitoring program. As above, these data were collected by taking depth profiles. To eliminate the daily fluctuation component, monitoring was always performed between 8:00 and 11:00 and the order of site monitoring kept consistent each week for the five sites in the bay and one site 2.5 km offshore (Colon; Figure 1). From this dataset, we extracted DO values at 1, 10, and 20 m depths at each site. Data were plotted to determine DO trends and seasonality patterns.

**Figure 2:**
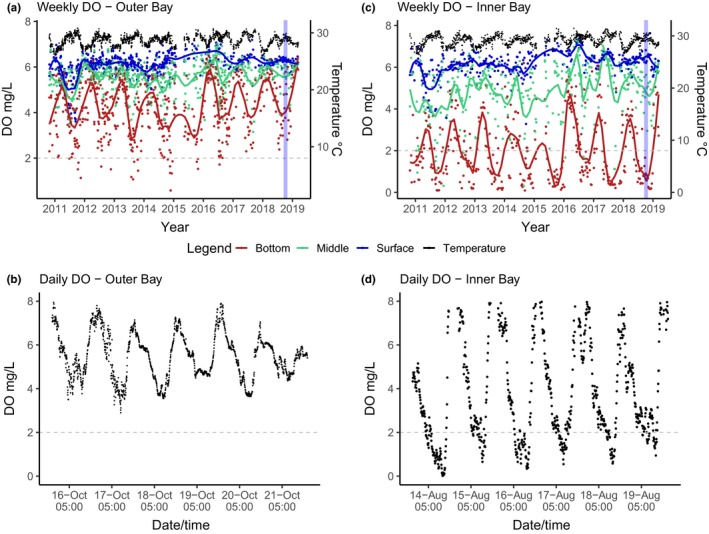
Variation in DO through time. Top: DO from 2010 to 2018 by depth recorded weekly at an (a) inner bay site (Alm) and a (b) outer bay site (HP). Colors indicate depth group (red: 20 m, green: 10 m, blue: 1 m), which are fitted with Loess smoother lines, whereas black points indicate temperature values across depths. Shaded band in 2018 indicates the period covered by the lower plots. Bottom: DO on patchy reef habitats at 3 m for 7 days (c) outer bay site (HPR) with a temperature range between 28.91–31.40°C and (d) inner bay site (TO) with a temperature range between 30.37–31–95°C. Data points below the gray dotted line at 2 mg/L indicate hypoxic conditions

To determine how DO conditions vary through the day–night cycle and how they differ between a shallow reef habitat in the most hypoxic part of the bay (Tierra Oscura: TO) and one in a less hypoxic area (Hospital Point Reef: HPR), we deployed an optical dissolved oxygen sensor on both reefs (MiniDOT, PME; YSI Pro2013). The sensors were deployed during the bay's most extreme hypoxic season, mounted on cement blocks, and elevated approx. 0.3 m above the seafloor at 3 m depth between March and November in 2018 (Figure 2; Table 1). Temperature and DO were recorded every 10 min. From these data, we determined how frequently, and for how long, each habitat experienced four different levels of oxygen conditions commonly associated with sublethal thresholds of benthic metazoans. These were dysoxia, 0.1–1.0 ml/L; hypoxia, >2 mg/L; moderate hypoxia, 2–5 mg/L; oxia, >5 mg/L (Vaquer‐Sunyer & Duarte, 2008). The frequency and duration of hypoxic conditions used in the fireworm experiment (below) were based on this continuous DO monitoring dataset from the site representative of the most intense hypoxic conditions in the bay, which showed that hypoxic conditions often occur twice a week for 8 hr.

**Table 1:** Descriptive statistics and relative frequencies of DO conditions in both the weekly and continuous monitoring surveys

	Site name & abbreviation	Depth (m)	Descriptive statistics DO mg/L				Relative frequency % time DO mg/L		
			Min	Med	Avg	Max	<2	2–5	>5
Weekly monitoring^a^	Colon	1	3.88	6.14	6.11	7.81	0.00	3.06	95.24
		10	3.41	5.87	5.80	6.97	0.00	6.09	93.91
		20	2.85	5.55	5.49	6.50	0.00	13.94	86.06
	Almirante (Alm)	1	0.41	6.22	6.13	7.52	0.32	4.81	89.42
		10	0.31	5.25	4.92	7.50	4.70	35.91	57.73
		20	0.09	1.80	2.11	6.20	53.06	41.67	5.28
	Pastores (Past)	1	3.86	6.25	6.21	7.97	0.00	4.00	90.30
		10	0.50	5.14	4.76	8.65	6.00	39.00	52.00
		20	0.09	1.76	2.12	6.31	53.13	43.87	3.00
	Hospital Point (HP)	1	3.61	6.13	6.09	7.64	0.00	3.76	95.06
		10	2.01	5.65	5.54	7.02	0.00	17.14	82.70
		20	0.58	4.40	4.28	6.49	3.51	61.71	34.78
	STRI	1	3.63	6.28	6.20	7.78	0.00	4.11	91.77
		10	2.96	5.90	5.71	7.07	0.00	16.62	82.83
		20	0.52	4.30	4.18	6.55	5.26	63.71	31.03
Continuous monitoring	Tierra Oscura (TO)^b^	3	0.02	5.17	5.28	14.76	2.92	42.67	39.63
	Hospital Point Reef (HPR)^c^	3	2.90	5.52	5.64	9.23	0.00	29.99	58.09

Note

Date range of data: ^a^2011–2018, ^b^12 March–1 November 2018 (234 days), ^c^15 October–15 November 2018 (31 days).

In all instances, DO measurements were corrected for pressure, salinity, and temperature.

### 2.3 Fireworm response to hypoxia

*Hermodice caranculata* was collected by hand from a patch reef dominated by *Porites* spp. (finger coral) and *Millepora alcicornis* (fire coral), near the STRI Bocas del Toro Research Station in Almirante Bay, Panama in February 2018 (MiAmbiente Scientific Research Permit #4149). A total of 24 worms were acclimated to an outdoor aquarium with running seawater for 5 days and then divided into two experimental treatments: 12 in constant normoxia (control) and 12 in fluctuating hypoxia (hypoxia). Three worms, each with unique color patterns, were assigned to four replicate 4 L tanks in each treatment. During the 8 hr exposure periods twice a week, seawater flow was stopped and hypoxic tanks were bubbled with pure N_2_ to achieve dissolved oxygen values of ~1 mg/L (~3.2 kPa), while control tanks were bubbled with ambient air to maintain normoxic DO values (6 mg/L; 19.5 kPa). DO levels were measured hourly using a handheld DO, Conductivity, Salinity instrument with a galvanic DO probe (YSI Pro2013). The average tank DO was 1.29 mg/L (±0.69) in the hypoxic treatment and 6.31 mg/L (±0.46) in the normoxic treatment. The low density of worms in the tanks, along with prior observations of worms without aeration indicated DO reductions during these exposures were primarily due to the nitrogen bubbling and not worm respiration. After exposure periods, flowing seawater was resumed and normoxic conditions maintained in all tanks. Temperature averaged 30.22°C (±0.53) in all tanks throughout the experiment. Each tank was cleaned weekly, and individuals were fed fish meat ad libitum once a week. The experiment ran for 8 weeks. One individual from the normoxic treatment escaped from its tank and died during the 6th week of the experiment, otherwise there was no mortality.

Resting respiration rates (MO_2_) were measured at the beginning of the experiment before any manipulation, then again after 4 and 8 weeks using an intermittent flow respirometry system (Q‐Box AQUA, Quibit Systems).Worms were removed from their exposure tanks (within the last 2 hr of an exposure period) and rinsed with 0.45 μm filtered seawater before being transferred to a cylindrical respirometry chamber (0.215 L) within a 15 L water bath containing filtered (0.45 μm), oxygenated seawater. The respirometry chamber was flushed with oxygenated seawater from the water bath to give the worm a resting period of approx. 10 min, or until the worm became still and chaetae were withdrawn. When the worm appeared to be in a resting state, the flow through the chamber was switched to a closed circulation loop for 5 min and the rate of oxygen consumption recorded. After 5 min, a value chosen because it corresponded to a decline in oxygen levels <80%; water flow was switched to introduce the oxygenated water from the bath for 3.3 min (200 s) to allow the worm to recover. This cycle was repeated up to six times per worm. A fiber optic dissolved oxygen probe within both the closed and open water circuits recorded oxygen levels every 15 s throughout the duration of the trial (approx. 30 min per worm). MO_2_ was calculated for each of the closed loop cycles using the following equation: MO_2_ = DO Slope (Vr − Va) * 3,600 m^−1^.

- MO_2_ oxygen consumption (mg kg^−1^ hr^−1^)
- DO slope rate of decrease of DO (mg L^−1^ s^−1^)
- Vr respirometer volume (L)
- Va volume of experimental animal (L)
- m animal weight (kg)

The individual's MO_2_ was averaged for the final resting respiration rate, which accounted for organism weight and volume. Background measurements of oxygen consumption from microbial activity were recorded before and after individual trials and found to be generally negligible. Water was changed and equipment cleaned between trials when background respiration rates were greater than (>5%). In the few occasions, when background respiration values were not negligible, it was usually due to worms spawning during the trial. In these cases, the worm was put back in the experimental tanks and respiration redone two days later. No worm was fed within 24 hr of respiration trials. An aquarium heater (Eheim JAGER thermocontrol 300 W precision heater, Germany) and circulation pump were used to keep temperature stable at 29°C for all trials.

After the 8‐week experiment, five individuals from each treatment were randomly chosen and exposed to 4 hr of hypoxia. The MO_2_ of these individuals was measured within 5 min after this exposure, and responses of the normoxic worms were compared to those from periodic hypoxia treatment. The number of individuals in this final exposure trial was limited by the number of respirometry runs that could be performed in a single day.

**Figure 3:**
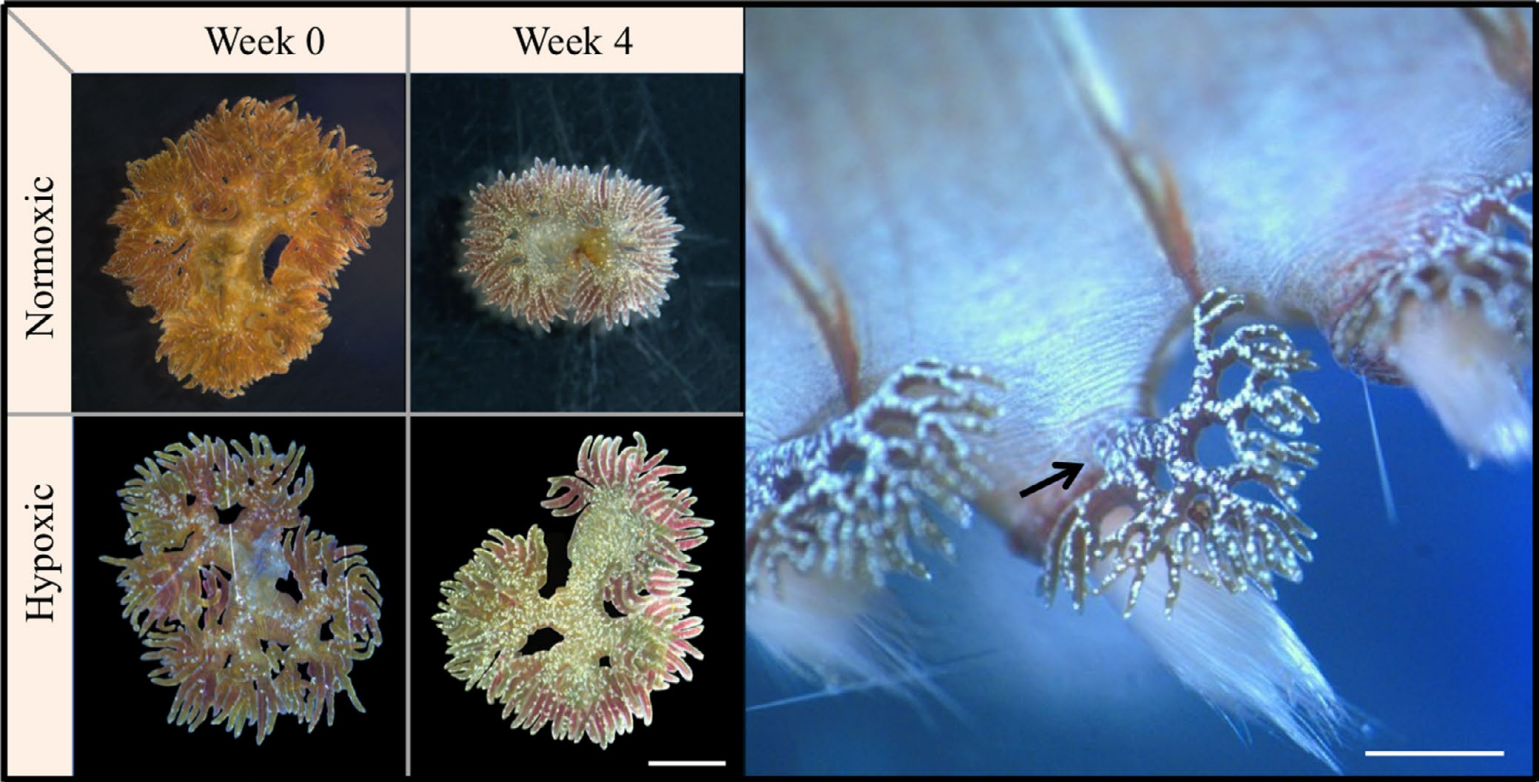
Left: Top panels illustrate the reduction of gill size in one individual from normoxic conditions after 4 weeks, and bottom panels illustrate the increase from one individual maintained in hypoxic conditions after 4 weeks (gills of the 11th left chaetiger in both worms). Right: Magnified image of gills, with the arrow indicating the position where gills were severed. Scale 1 mm

Immediately after an individual's MO_2_ was measured, its gill morphology, wet weight and volume were measured. Worms were gently blotted with a towel and weighed and then put in a beaker with seawater to measure their volume by water displacement. Each worm was then anaesthetized in 10% MgCl. After ~15 min, worms were placed under a dissecting microscope. We measured the surface area of the gills to determine the number of gill filaments that regrew during the experiment: the left top gill on the 11th chaetiger was removed at the base using fine tip tweezers (Figure 3). The severed gill was immediately placed on a glass slide with cover slip and photographed with a digital camera (Nikon Sight DS‐U1, Nikon, Milan, Italy). The gills on the left of the 11th chaetiger of every worm were removed and measured at the start of the experiment prior to any experimental manipulation. On week 4 and 8, they were removed again from the same location and the regrown gills measured. We only severed the gills at this one position to avoid undue stress on the organism. Surface areas (mm^2^) were measured from the photographs of the severed gills by tracing the gill filaments and analyzing the area with ImageJ software, and gill filaments were counted from the photographs (Rasband WS, US National Institutes of Health) (Abràmoff, Magalhães, & Ram, 2004). We attempted to count filament tips from nonsevered gills and found counts to be difficult and inaccurate due to short anesthetic time and three‐dimensional gill morphology, thus only present our results from severed gill measurements.

A 2‐way repeated measures ANOVA was used to test the importance of DO treatment, week, and their interaction on (a) worm wet weight, (b) regenerated gill surface area, (c) regenerated gill filament number, and (4) mass adjusted respiration rates. We present both gill morphology traits (area and filament counts) to show the utility in both measures as a means to study annelid gill morphology in living animals. Additionally, wet weight was used to assess worm growth throughout the experiment instead of chaetigers because preliminary data exploration showed differences with wet weight, but not chaetigers.We analyzed the results from the final respiration trial (after the short “DO_stress” test subsequent to the week 8 experiment) separately, with ANOVA, to determine whether there was an effect of treatment on respiration rates after DO exposure. In all cases, residuals were assessed for normality by building histograms, and they were checked for nonlinearity, unequal error variances, and outliers by plotting the residuals against fitted values (Zuur, Ieno, & Elphick, 2010).

All statistical analyses were performed by using the statistical software R (v.3.5.1; R Core Team 2018). All physical monitoring data and fireworm experimental data are available in the Dryad repository: https://doi.org/10.5061/dryad.c2fqz614c.

## 3 RESULTS

### 3.1 Spatiotemporal variation in environmental oxygen concentrations

Hypoxic conditions are consistently most severe close to the mainland (herein referred to as the inner bay) compared to the areas around the outer islands (i.e., Figure 1; outer bay). This is supported by both the spatial analysis from the September 2017 survey and the 8 years of monitoring data. DO is also consistently lower at depth (i.e., at 20 m compared to 10 m) throughout the bay (Figure 1, Table 1).

There is also strong seasonal variation in DO, with hypoxia typically occurring throughout the second half of the year from July to December (Figure 2). The beginning and end of the hypoxic season vary by 1–2 months depending on the year. This seasonal pattern was most pronounced at 20 m, in both the inner and outer bay sites. At 10 m, the seasonal pattern was evident in the inner bay but not in the outer bay. No seasonal pattern was detected in the surface in either inner or outer bay sites.

During the hypoxic season, DO at the two 3 m sites with continuous monitoring both exhibited diurnal cycles (Figure 2, Table 1). The inner bay had larger DO fluctuations (TO: 0.02–14.76 mg/L/ 0.055–47.586 kPa) compared to the outer bay (HPR: 2.90–9.32 mg/L/ 9.583–29.900 kPa). During the continuous monitoring, anoxic conditions never occurred in HPR (outer bay) and conditions were normoxic for the majority of time (58%). In TO (inner bay), anoxic conditions occurred 3% of the time, with an average duration during the 24‐hr day cycles of 8 hr (<1 mg/L). The lowest DO levels mostly occurred between 4 and 8 a.m. These low DO levels were also more erratic/unpredictable, as anoxia did not occur every morning—in some instances, it occurred consecutively for 5 days and then did not occur again for 2 weeks. The average number of days when anoxic conditions occurred during the hypoxic season at TO (inner bay) was approximately twice per week during the monitoring period/hypoxic season, between 21 July and 7 October.

### 3.2 Fireworm response to hypoxia

**Figure 4:**
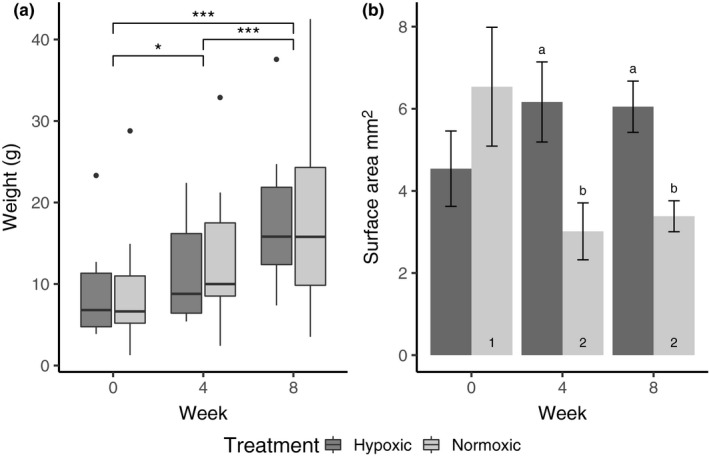
(a) Weekly wet weight of worms from hypoxic and normoxic treatment conditions, colored in black and gray, respectively, during the 8‐week experiment. Significant differences between weeks indicated by asterisks. (b) Change in mean number of regenerated gill surface area (±*SE*) on the 11th left cheatager, from hypoxic and normoxic treatment conditions. Significantly different mean values (*p* < .05) at different DO treatments for the same week are indicated by different letters placed above the histograms, while significantly different mean values (*p* < .05) within the same DO treatment among different weeks are indicated by different numbers placed inside the histograms. Pairwise comparisons were conducted using the Estimated Marginal Means test with Least Significant Difference test correction

The worms gained significant weight throughout the 8‐week experiment. The average worm weight at the beginning of the experiment was 9 g for both treatments (hypoxic: 9.5 g; normoxic: 9.2 g), and after eight weeks, weights significantly increased by over 100% to an average of 18.3 g in both treatments (hypoxic: 17.6 g; normoxic: 19.1 g; week: *p* > .001 Figure 4a), with no difference between treatment group.

Despite similar worm growth in both treatments throughout the experiment, the change in the area of the regenerated gills (and number of regenerated gill filaments) from the beginning to the end of the experiment was significantly effected by DO treatment (treatment * week interaction: area: *p* = .005, filaments: *p* = .003, Figures 3 and 4b, Table 2). The regenerated gill area of worms from the normoxic treatment on week 4 was 54% smaller than their original gill area before being severed (week 0:6.54 ± 1.45 *SE*; week 4:3.01 ± 0.69 *SE *mm^2^). After being re‐severed on week 4, normoxic worms regrew their gills to approximately the same size as in the first 4 weeks of the experiment (week 8:3.38 ± 0.37 *SE *mm^2^). This is in contrast to the regenerated gills of worms in the hypoxic treatment that were on average 33% bigger after both 4 and 8 weeks, than those initially severed on week 0 (week 0:4.54 ± 0.92 *SE *mm^2^; week 4:6.17 ± 0.97 *SE *mm^2^; week 8:6.05 ± 0.62 *SE *mm^2^). On both week 4 and 8, there were differences between the DO treatments, with gill areas 51% and 44% smaller in normoxic worms compared to hypoxic worms, respectively (Figure 4b). As in gill surface area, the number of regenerated filaments on normoxic worms' gills on week 4 was 44% less than those on their gills initially: week 0:103 ± 20.9 *SE* to week 4:58 ± 9.3 *SE* filaments. Gill filament counts on week 4 and 8 were similar, with 35% fewer filaments on week 8 compared to those on the initial unsevered gills (week 8:67 ± 8.09 *SE* filaments). In hypoxia, there were 24.3% more filaments in the regenerated gills throughout the experiment;: week 1:64 ± 7.9 *SE*, week 4:86 ± 10.6 *SE*, week 8:80 ± 8.4 *SE* filaments (Table 2).

**Table 2:** Results of 2‐way repeated measures ANOVAs investigating the effect of DO treatment (hypoxic or normoxic) on 1) worm weight, 2) gill surface area, and 3) respiration rates (MO_2_) through time (weeks) in the fireworm *H. caronculata*

Predictor	*df*_Num_	*df*_Den_	Epsilon	SS_Num_	SS_Den_	*F*	*p*	$\eta_{g}^{2}$
1. Total worm wet weight (g)								
(Intercept)	1.00	22.00		12,152.79	3,985.70	67.08	.000	0.73
Treatment	1.00	22.00		10.64	3,985.70	0.06	.811	0.00
**Week**	**1.84**	**40.41**	**0.92**	**860.95**	**479.35**	**39.51**	**.000**	**0.16**
Treatment × Week	1.84	40.41	0.92	10.62	479.35	0.49	.602	0.00
2. Gill surface area (mm^2^)								
(Intercept)	1.00	22.00		176,304.18	33,208.79	116.80	.000	0.73
Treatment	1.00	22.00		2,922.94	33,208.79	1.94	.178	0.04
Week	1.65	36.20	0.82	1,274.31	31,465.84	0.89	.401	0.02
**Treatment × Week**	**1.65**	**36.20**	**0.82**	**9,695.79**	**31,465.84**	**6.78**	**.005**	**0.13**
3. Gill filament counts (#)								
(Intercept)	1.00	22.00		418,765.01	67,681.64	136.12	.000	0.79
Treatment	1.00	22.00		5.01	67,681.64	0.00	.968	0.00
Week	1.65	36.39	0.83	2,025.03	42,517.11	1.05	.349	0.02
**Treatment × Week**	**1.65**	**36.39**	**0.83**	**14,881.19**	**42,517.11**	**7.70**	**.003**	**0.12**
4. Respiration rate (MO_2_ mg kg^−1^ hr^−1^)								
(Intercept)	1.00	22.00		1,908,374	309,571	135.62	.000	0.80
Treatment	1.00	22.00		6,596	309,571	0.47	.501	0.01
Week	1.53	33.76	0.77	15,002	170,353	1.94	.168	0.03
Treatment × Week	1.53	33.76	0.77	10,464	170,353	1.35	.268	0.02

Note

Significant effects are in bold. *df*_Num_ indicates degrees of freedom numerator. *df*_Den_ indicates degrees of freedom denominator. Epsilon indicates Greenhouse–Geisser multiplier for degrees of freedom, *p*‐values and degrees of freedom in the table incorporate this correction. SS_Num_ indicates sum of squares numerator. SS_Den_ indicates sum of squares denominator. $\eta_{g}^{2}$ indicates generalized eta‐squared. Bold font highlights the variables that have a statistical significance (*p* ≤ 0.005).

**Figure 5:**
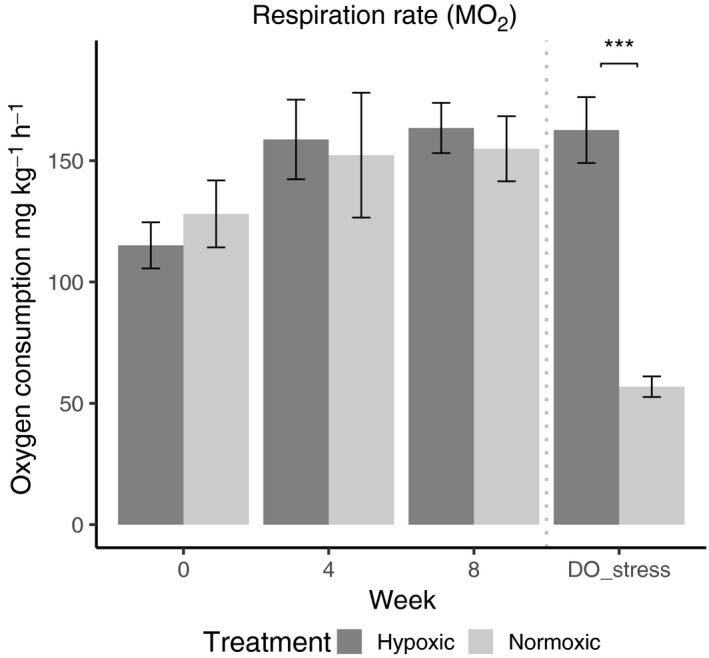
Average respiration rates of worms from hypoxic and normoxic DO treatments initially (week 0—no DO exposure), and mid‐experiment (week 4), and at the end (week 8); black and gray bars, respectively. The DO stress trial results are respiration rates post‐4 hr hypoxia exposure in both treatments after 8 weeks, indicated by “DO_stress.” Error bars show *SE*, and asterisks indicate a significant difference between treatments (*p* > .005)

Analysis of respiration rates during the experiment showed there was no significant effect of week or treatment, or of the interaction between the two (Figure 5, Table 3). At the end of the experiment after the hypoxia “stress” trial, experimental treatment had a significant effect on respiration rate (*p* = .005, Table 3). The worms that were maintained in normoxic conditions for 8 weeks demonstrated a dramatic decline in respiratory rate after being exposed to 4 hr of hypoxia – from 154.89 (before DO stress) to 56.84 mg kg^−1^ hr^−1^ (after DO stress) a 63% decrease (Figure 5). This respiratory response of the normoxic worms after the DO stress was significantly lower than any other respiratory response in either treatments, throughout the entire experiment. In contrast, worms from the hypoxic treatment demonstrated a very different response, maintaining the same stable oxygen consumption rate as in week 8, when they were tested after their last exposure period (164 mg kg^−1 ^hr^−1^ after experimental week 8–163 mg kg^−1 ^hr^−1^ after end DO stress trial).

**Table 3:** ANCOVA results investigating MO_2_ after the hypoxic “stress” exposure on worms from both treatments (hypoxic or normoxic) after the 8‐week experiment, with worm weight as a covariate

Predictor	Sum of squares	*df*	Mean square	*F*	*p*
(Intercept)	79,707.96	1	79,707.96	45.19	.000
**Treatment**	**18,593.36**	**1**	**18,593.36**	**10.54**	**.005**
Size (weight)	2,810.19	1	2,810.19	1.59	.224
Error	29,983.58	17	1,763.74		

Note

Bold font highlights the variables that have a statistical significance (*p* ≤ 0.005).

**Figure 6:**
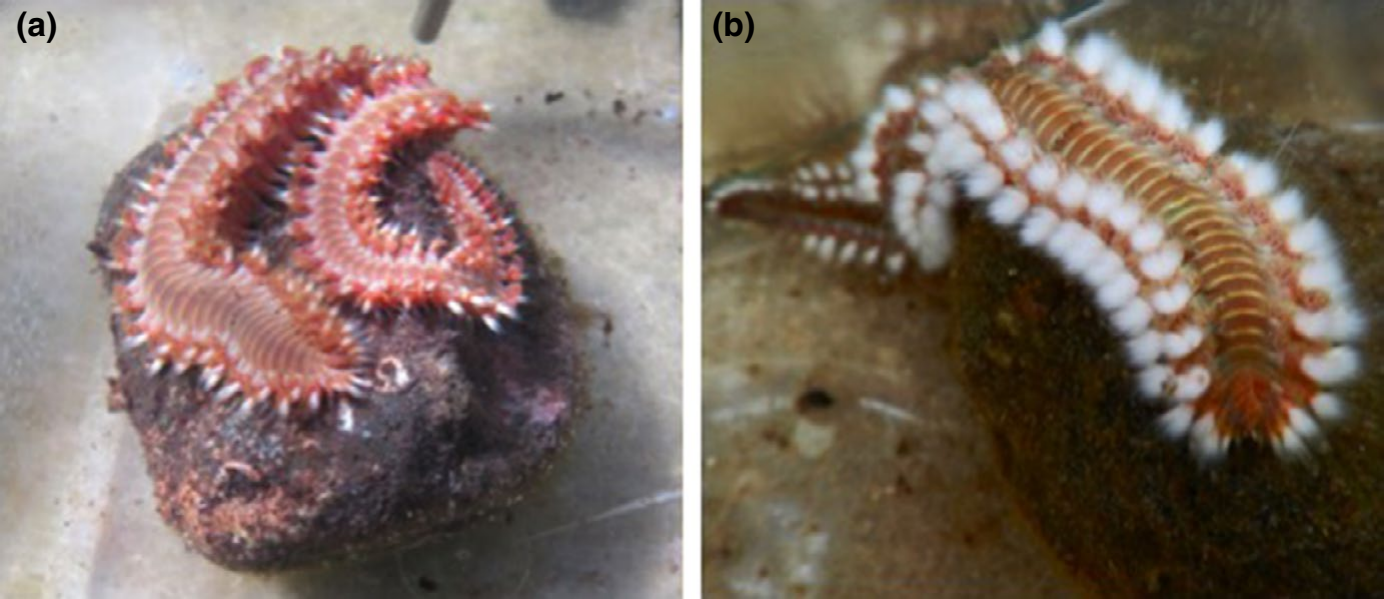
(a) Fireworm *Hermodice caranculata* exhibiting behavioral response observed in worms subjected to hypoxia; where they climb to the highest point in tank and lay motionless/unresponsive, (b) active worm in normoxic conditions with flared harpoon chaetae

The experimental worms also showed distinct behavioral responses to the treatments. As the hypoxia treatment progressed many of the worms exhibited avoidance and energy conservation behaviors. During hypoxia exposure periods, they would perch themselves on top of the rock in the tank and remain motionless for the rest of the exposure. Others would prop themselves against the side of the tank and stay still in that elevated position, or attempt to push the tank lids off and climb out. When touched in hypoxia, they would not respond with the typical flare of their chaetae (Figure 6).

## 4 DISCUSSION

In Bahia Almirante, we found a clear seasonal pattern of hypoxia. The weekly measurements over eight years demonstrated strong seasonality with hypoxia occurring throughout the bay between July and December. This hypoxia may be due to a combination of influences including high biological productivity (Kinsey & Kinsey, 1967; Wild et al., 2010), geographically limited water circulation and protection from wind‐driven mixing (Li & Reidenbach, 2014), stratification and warmer temperatures (Altieri & Gedan, 2015), or large‐scale climate patterns (Collin, D'Croz, Gondola, & Del Rosario, 2009)—all of which are known to promote conditions favorable for hypoxia formation. The spatial survey also shows anoxic or severe hypoxic conditions in the inner bay, while the outer bay is less hypoxic and less stratified. As many species, including fireworms, are distributed throughout the bay, they must employ strategies to cope with either persistent or periodic hypoxia in all but the most exposed and well‐flushed sites.

One of the most interesting findings of this study was that the strong spatial hypoxic gradient documented at depth is reflected in adjacent shallow water as the magnitude of diurnal DO change. Sites experiencing chronically low DO at 20 m were affiliated with shallow sites having the greatest diurnal DO fluctuations, ranging from anoxia at night to supersaturation during the day. This was also related to extreme DO fluctuations occurring more often and anoxic conditions lasting for longer. Some evidence of this type of DO variability has been shown in other highly productive biological reef habitats (>5 m) (Kinsey & Kinsey, 1967; Niggl, Haas, & Wild, 2010; Wild et al., 2010), but such variation is not well documented and it is seldom taken into account in experimental designs. This is pertinent as such shallow regions are where the most biodiverse seagrass and coral reef habitats occur, indicating that the associated organisms regularly experience periods of hypoxia and/or anoxia (Nilsson, Östlund‐Nilsson, & Munday, 2010; Nilsson & Ostlund‐Nisson, 2003).

By replicating the frequency, magnitude, and duration of DO in the shallow hypoxic areas of the bay in the laboratory, we were able to test whether and how the corallivourous fireworm *H. carunculata* from more normoxic reef habitats respond to the conditions found at sites experiencing more frequent, periodic hypoxia. We predicted that worms in hypoxic conditions would regenerate larger gills, compared to worms in normoxia. *Hermodice carunculata* did regenerate larger gill structures in hypoxic environments (Figure 3). Similar gill regeneration has been noted in other annelids to compensate for losses in gas exchange capacity after predation of segments (Zoran, 2010). However, here the worms in normoxic conditions had an unexpected response in that they regenerated fewer and smaller gill structures than they had when initially taken from the field (Figure 4). This response occurred independently of the worm's weight change, which increased at the same rate for both treatments. The most plausible explanation for this is that the worms from the presumed normoxic reefs are actually experiencing more hypoxia in situ than in our experimental normoxic treatments. They are likely exposed to hypoxia in burrows or reef crevices even in well‐oxygenated waters, due to low oxygen conditions common in these microhabitats and in the boundary layers on reefs (Wong et al., 2018). Moreover, they are known to be nocturnal and most active from dawn to dusk when oxygen levels are the lowest on these shallow reefs (Schulze, Grimes, & Rudek, 2017). Worms that do not experience hypoxia pressures (i.e., those in the normoxic treatment) may not be investing as much energy in gill regeneration because there is not a strong need to. A slower rate of gill regeneration due to lower environmental pressure could explain why the hypoxic worms regrew their gill structures quickly while normoxic worms did not. Whatever the driving factor, these results support the idea that gill generation is mediated by environmental oxygen levels and that in experimentally well‐oxygenated conditions, worms will spend less effort on gill maintenance (Hervant, Mathieu, & Messana, 1998).

It is likely that changes in gill morphology correspond to the worms' ability to increase oxygen uptake. Under hypoxia, most invertebrates are expected to have reduced oxygen consumption (Grieshaber, Hardewig, Kreutzer, & Pörtner, 1994), and new gill growth may facilitate more efficient oxygen consumption either during or after hypoxia exposure (Kristensen, 1983). Here, we measured respiration rates after hypoxia exposures. Upon reoxygenation of hypoxic water, there is a well‐known respiratory occurrence in invertebrates where they consume a supernormal amount of oxygen to restore an oxygen debt acquired while in hypoxia. This increase in oxygen use is considered an important functional part of metabolic recovery following anaerobic metabolism in many invertebrates (Ellington, 1983). It allows the organism to dispose of anaerobic end products and resaturate the body tissues with oxygen (Bridges & Brand, 1980). We therefore expected to see higher respiration rates upon reoxygenation, which could be attributed to the repayment of an oxygen debt (Bennett, 2017; Sander, 1973). As in fireworms tested under high temperatures (Ferraris, 1981), respiration rates during this experiment were similar regardless of treatment, with no evidence of oxygen debt payments in the hypoxic treatment (Figure 5). This lack of oxygen debt combined with greater gill regeneration capabilities indicates higher functionality of the respiratory structures during hypoxia (Sander, 1973). With highly effective gills able to absorb even the smallest amounts of oxygen in the environment, aerobic metabolism may be continuing even under very low oxygen levels. This could explain the lack of oxygen debt seen in the hypoxic treatment; however, this possibility needs further investigation.

One of the most striking results found here was that the worms maintained in normoxic conditions for 8 weeks and then exposed to a 4 hr hypoxia stress, demonstrated dramatically depressed respiration rates upon reoxygenation. These worms seemed to be experiencing metabolic depression, while worms with a history of hypoxic exposure did not. Many instances of oxygen debt in invertebrates have shown highly variable responses and have been explained by the varying abilities of different species to reduce the rate of aerobic metabolism under anoxic conditions to avoid anaerobic byproducts (Herreid, 1980). However, a few studies have demonstrated the intraspecific differences seen here. Hypoxic conditioning in this study appears to give the worms an advantage through prompt compensation of metabolic depression occurring during hypoxia exposure (Figure 5; no noticeable oxygen debt). It suggests that by switching metabolic efforts on and off in response to oxygen availability they may be able to take advantage of oxygen when it is present in the environment—a trait of great functional importance in rapidly changing environments (Ellington, 1983; Herreid, 1980). These responses are likely acting synergistically with other physiological responses to facilitate recovery between hypoxic periods (Borowiec et al., 2015), and would be better explained by fine‐scale temporal studies on intermittent hypoxia responses.

In addition to this metabolic plasticity with respect to hypoxia recovery, the worms also showed distinct behavioral responses to the treatments. It seemed as though they had an innate response to move closer to the surface by elevating themselves in the tank, which in nature would correspond with moving to more oxygenated waters (Figure 6). These behaviors are similar to hypoxia avoidance responses in other species in hypoxic systems such as copepods and blue crabs (Bell, Eggleston, & Wolcott, 2003; Decker, Breitburg, & Marcus, 2003). One potential ecological consequence of this behavior is that the worms may be subjecting themselves to greater predation risk by leaving the protection of reef crevices. Some of the notable fireworm predators are reef fish (white grunt, *Haemulon plumierii*; sand tilefish, *Malacanthus plumieri*; whitebone porgies*, Calamus leucosteus*) (Ladd & Shantz, 2016; Sedberry, 1989), and evidence suggests that reef fish may have quite high hypoxia tolerances (Nilsson & Ostlund‐Nisson, 2003; Wong et al., 2018). Identifying mismatches of hypoxia tolerance between predator and prey behavior in a rapidly changing oxygen environment would be an interesting avenue to understand the longer‐term ecosystem consequences of hypoxia on coral reefs (Riedel et al., 2014).

Our results suggest that *H. carunculata* has the physiological ability to maintain and withstand hypoxia without high cost to the individual. Fireworms may therefore have an advantage as environmental imbalances such as hypoxia alter ecosystem function (Schulze et al., 2017). These worms are known to thrive in degraded areas, prey on live coral and their recruits (Nicolet, Chong‐Seng, Pratchett, Willis, & Hoogenboom, 2018), preferring weakened or stressed corals (Wolf et al., 2014) and are also known to be vectors for diseases involved in coral bleaching (Williams, 2007). In addition, *H. carunculata* is on the brink of being considered an invasive pest species with population sizes rapidly increasing in some areas (Williams, 2007; Simonini, Righi, Maletti, Fai, & Prevedelli, 2017). The interactive effects of increased anthropogenic pressures, including deoxygenation, and weakened corals attracting more corallivory have negative ecological implications, and the potential to be realized throughout this fireworm species' circumtropical Atlantic–Mediterranean range (Rice, Ezzat, & Burkepile, 2019; Simonini, Maletti, Righi, Fai, & Prevedelli, 2018).

## 5 CONCLUSIONS

Our results indicate that hypoxic conditions in shallow reefs may be underestimated due their daily variability, with minimum levels occurring at night, when manual measurements are usually lacking. We show that the fireworm is able to tolerate extreme oxygen conditions and that this species will be able to survive potentially worsening conditions. However, it should be emphasized that future deoxygenation may exceed the ability of the worms to compensate (Ashander, Chevin, & Baskett, 2016; Hameau, Mignot, & Joos, 2019; Nilsson et al., 2010). Future research needs to tease apart organismal responses to natural DO variability and increasingly anthropogenic driven DO change, to determine the implications of these processes for reef invertebrates and ecosystems. Filling this knowledge gap is imperative for tropical marine benthos that will be most vulnerable in the face of future human‐driven changes.

## CONFLICT OF INTEREST

None declared.

## AUTHOR CONTRIBUTIONS

All authors helped design the experiment; NML and MC performed the experiment. All authors contributed to the analysis and writing of the manuscript.

### OPEN RESEARCH BADGES

This article has earned an Open Data Badge for making publicly available the digitally‐shareable data necessary to reproduce the reported results. The data is available at https://doi.org/10.5061/dryad.c2fqz614c

## Data Availability

Physical monitoring data and fireworm experimental data are available in the Dryad repository: https://doi.org/10.5061/dryad.c2fqz614c
